# Relationship of sleep disorders with long-term complications and health-related quality of life in people with well-controlled human immunodeficiency virus

**DOI:** 10.1097/MD.0000000000029070

**Published:** 2022-03-18

**Authors:** Yusuke Yoshino, Yoshitaka Wakabayashi, Takatoshi Kitazawa

**Affiliations:** ^a^ *Department of Microbiology, Teikyo University School of Medicine, 2-11-1, Kaga, Itabashi, Tokyo, Japan,* ^b^ *Department of Internal Medicine, Teikyo University School of Medicine, 2-11-1, Kaga, Itabashi, Tokyo, Japan.*

**Keywords:** human immunodeficiency virus, health-related quality of life, long-term complication, sleep disorder

## Abstract

Although sleep disorders are common in patients with human immunodeficiency virus (HIV) infection, they have not been adequately evaluated under currently advanced treatments, mainly with integrase strand transfer inhibitors. However, the relationship of sleep disorders with long-term complications and quality of life (QOL) status in patients infected with HIV is still poorly understood. Such associations are important in the management of outpatients with HIV. Hence, this study aimed to evaluate these associations.

This cross-sectional observational study assessed the QOL changes of patients with HIV before and after the treatment regimen change. Male patients with well-controlled HIV who attended our hospital and changed HIV medications for reasons other than treatment failure between October 2019 and September 2021 were included. At the time of regimen change, sleep disorder status was assessed according to the Pittsburgh sleep quality index (PSQI), and health-related QOL (HRQOL) was assessed using the medical outcomes study 8-item short form health survey. In addition, we collected information on age, blood tests, and long-term comorbid conditions present during the evaluation. The HIV treatment regimen was also reviewed.

Out of 45 male Japanese patients with HIV that were included in this study, 24 (53.3%) and 21 (46.7%) were classified into the sleep disorder group and nonsleep disorder group, respectively, according to their PSQI scores. The sleep disorder group had a significantly lower HRQOL mental component summary (*P* = .0222) than the nonsleep disorder group. The prevalence rates of hypertension, dyslipidemia, and diabetes mellitus were not significantly different between the 2 groups. In addition, a significant correlation was observed between PSQI scores and the HRQOL status (mental component summary, *P* = .0450; physical component summary, *P* = .0350).

Sleep disorders remain common in patients with well-controlled HIV infection receiving current treatment. Sleep disorder is significantly associated with a low HRQOL in these patients. Hence, sleep status evaluation is necessary to improve HIV management.

## 1. Introduction

Sleep is one of the most important factors influencing one’s performance. The quality and quantity of sleep affect a wide range of characteristics, from physical and mental performance to general health. Regarding physical performance, sleep significantly contributes to muscle recovery and restoration.^[1]^ Sleep is also closely related to physical and mental health. In fact, it has been associated with depression, anxiety, and bipolar disorder.^[2]^

Sleep disorders are common in people living with human immunodeficiency virus (HIV) infection (PLWH).^[3]^ However, the complication rate of sleep disorders remains controversial. Sleep disorders are often evaluated subjectively, making them difficult to diagnose. In recent years, objective measures have been used to evaluate sleep disorders. For instance, Pittsburgh sleep quality index (PSQI) is a comprehensive assessment method based on not only sleep duration but also other sleep parameters, such as sleep efficiency, sleep latency, sleep quality, sleep disturbance, hypnotic medication, and daytime dysfunction.^[4]^ Many PSQI-based studies have also reported that sleep disorders are common in PLWH.^[5-14]^ Some of these studies have suggested that sleep disorders reduce the quality of life (QOL) of PLWH.^[7,15]^

Social background, HIV infection itself, and immune status alteration are the reported factors associated with sleep disorders in HIV infection.^[3]^ Previously, antiretrovirals, such as non-nucleoside reverse transcriptase inhibitors, were often associated with sleep disorders.^[10,11]^ However, considering the significant advances in HIV treatment, the use of non-nucleoside reverse transcriptase inhibitors, which have been linked to sleep disorders as adverse events, has been drastically reduced in recent years, and safer drugs with fewer adverse events are now being prescribed. In addition, the status of sleep disorders in well-controlled PLWH treated with recent safer drugs is still insufficiently evaluated.

The life expectancy of PLWH has dramatically increased, owing to the advancement of therapeutic drugs; as a result, lifestyle-related diseases known as long-term complications occur more frequently in PLWH than in people without HIV infection.^[16]^ Some of these long-term complications include hypertension, dyslipidemia, and diabetes mellitus. The management of these diseases has become important in the outpatient management of PLWH. In addition, regardless of the HIV infection status, the long-term complications of HIV infection are generally associated with sleep disorders, even in younger ones.^[17,18]^ Hence, HIV infection, sleep disorders, and long-term complications may be closely related to each other.

This study aimed to evaluate the actual status of sleep disorder and its association with long-term complications and other parameters in PLWH (virologically stable) treated with currently advanced antiretrovirals. We also examined their health-related QOL (HRQOL) according to patient-reported outcomes to determine the association between sleep disturbance and QOL in well-controlled PLWH.

## 2. Methods

This cross-sectional observational study, which was approved by the ethics committee of Teikyo University (No. TUIC-COI.19-175), assessed all patients who participated in a clinical study on QOL changes before and after treatment regimen modification. Male PLWHs who attended our hospital and changed their antiretrovirals medications for reasons other than treatment failure between October 2019 and September 2021 were enrolled in the study. The reason for the change in antiretrovirals medication is to remove the booster drug (emtricitabine) and to change to a single-tablet regimen. There were no cases of change due to adverse drug reactions. In particular, only well-controlled cases under anti-HIV treatment for at least 6 months and maintaining an HIV viral load of less than 200 copies/μL, were included.

At the time of regimen change, we assessed the participants’ sleep disorder status and HRQOL using the PSQI and the Japanese version of medical outcomes study 8-item short form health survey (SF-8), respectively. Both assessments were conducted using a questionnaire. In addition, we collected information on age, blood tests (white blood cell, hemoglobin, alanine aminotransferase, bilirubin aspartate aminotransferase, urea nitrogen, creatinine, and CD4-positive lymphocyte count), and long-term comorbid conditions present during the evaluation. The HIV treatment regimen was also reviewed.

According to the PSQI scores (sleep disorder: PSQI > 5), the patients were classified into 2 groups: sleep disorder group and nonsleep disorder group.

The 2 groups were assessed for differences in the presence or absence of complications, significant differences between each parameter, and significant differences in HRQOL. As mentioned, HRQOL was assessed with the Japanese version of SF-8.^[19]^ The SF-8 is an 8-scale HRQOL assessment tool that generates 2 summary measures: a physical component summary and a mental component summary (MCS). The higher the score, the better the HRQOL status. For discontinuous parameters, the significant factors related to sleep disorders were identified using the factorial square test or Fisher exact test. Fisher exact test does not rely on distributional assumptions; therefore, it is particularly appropriate for the analysis of small samples. Furthermore, the direct association of sleep disorder with PSQI values was evaluated using correlation analysis. StatFlex version 7 (Artech Co., Osaka, Japan) was used for all statistical analyses.

## 3. Results

This study included 45 male PLWHs. Table 1 presents the clinical features of patients classified into sleep disorder and nonsleep disorder groups according to their PSQI scores. Age, treatment duration, time since diagnosis, CD4 levels, and the HIV medication type were not significantly different between the 2 groups. The prevalence rates of hypertension, dyslipidemia, and diabetes mellitus also showed no significant differences between such groups. In the HRQOL measured by the SF-8, the MCS was significantly lower in the sleep disorder group than in the nonsleep disorder group.

**Table 1:** Clinical features of 2 groups of people living with HIV: sleep disorder group and nonsleep disorder group.

***n*** **= 45**		**all (*n* = 45)**	**Sleep disorder (+) (*n* = 24)**	**Sleep disorder (-) (*n* = 21)**	***P***
Age		45.0 (38.0-63.3)	45.0 (38.0-65.0)	45.0 (37.3-60.8)	.9406
Treatment duration (in wk)		79.0 (56.5-108.3)	78.0 (56.5-97.3)	83.0 (50.0-114.5)	.7896
Duration (in wk) after diagnosis		89.0 (57.8-112.8)	90.0 (58.5-104.3)	84.5 (50.5-130.5)	.6652
CD4-positive lymphocyte		599.0 (427.3-700.5)	587.5 (433.5-801.0)	639.0 (419.8-672.5)	.4127
White blood cell		5300.0 (4400.0-5925.0)	4250.0 (4500.0-5950.0)	5300.0 (4400.0-5925.0)	.7027
Hemoglobin		14.9 (14.4-15.6)	15.0 (14.5-15.8)	14.8 (14.3-15.5)	.8911
Total bilirubin		.550 (.475-.795)	.580 (.470-.800)	.540 (.473-.753)	.5527
Aspartate aminotransferase		21.0 (16.0-25.0)	24.0 (20.0-29.0)	18.0 (14.8-20.3)	.0778
Alanine aminotransferase		21.0 (14.0-34.3)	26.0 (18.5-40.0)	14.0 (11.5-21.0)	.1132
Blood urea nitrogen		14.6 (12.6-18.4)	14.86 (12.9-17.7)	13.9 (12.1-18.4)	.6854
Creatinine		.980 (.848-1.043)	.985 (.850-1.100)	.950 (.845-1.013)	.3892
HIV treatment regimen	
	INSTI use	35 (77.8%)	19 (79.2%)	16 (76.2%)	1.0000
	Second-generation INSTI use	15 (33.3%)	8 (33.3%)	7 (33.3%)	1.0000
	Single tablet regimen	31 (68.9%)	15 (62.5%)	16 (76.2%)	1.0000
	Booster use	28 (62.2%)	14 (58.3%)	14 (66.7%)	.8107
Comorbidities	
	Hypertension	14 (31.1%)	9 (37.5%)	5 (23.8%)	.5651
Dyslipidemia		15 (33.3%)	8 (33.3%)	7 (33.3%)	1.0000
	Diabetes mellitus	4 (8.9%)	2 (8.3%)	2 (9.5%)	1.0000
Health-related quality of life	
	Physical component summary	51.7 (48.1-54.2)	50.7 (47.1-54.3)	53.6 (49.3-54.2)	.1907
	Mental component summary	50.9 (48.1-54.2)	48.5 (42.2-51.7)	53.2 (49.3-54.6)	.0222
HIV = human immunodeficiency virus, INSTI = integrase strand transfer inhibitor.					

In the correlation analysis between the PSQI score and each item, a significant inverse correlation was observed between HRQOL, MCS, and physical component summary (Table 2).

**Table 2:** Correlation analysis between the PSQI score and each item.

		***R***	***P***
Age		.0728	.6347
Treatment duration (in wk)		.0136	.9292
Duration (in wk) after diagnosis		.0468	.7604
CD4-positive lymphocyte		.0047	.9756
White blood cell		-.1254	.4117
Hemoglobin		-.0791	.6054
Total bilirubin		.0679	.6577
Aspartate aminotransferase		.2922	.0514
Alanine aminotransferase		.1493	.3277
Blood urea nitrogen		.0352	.8186
Creatinine		.0938	.5401
HIV treatment regimen	
	INSTI use	.0430	.779
	Second-generation INSTI use	-.1012	.508
	Single tablet regimen	-.1082	.479
	Booster use	.0098	.949
Comorbidities	
	Hypertension	.1984	.191
	Dyslipidemia	.1984	.191
	Diabetes mellitus	.1341	.380
Health-related quality of life	
	Physical component summary	-.3151	.035
	Mental component summary	-.3005	.045
HIV = human immunodeficiency virus, INSTI = integrase strand transfer inhibitor, PSQI = Pittsburgh sleep quality index, *R* = correlation coefficient.			

## 4. Discussion

In this study, more than half of the Japanese patients with wellcontrolled HIV infection suffered from sleep disorders, consistent with the results of some previous reports.^[5-8,11,20]^ Hence, the sleep disorder rate was high, even in the setting of recent treatment mainly with integrase inhibitors and in a virologically stable state.

Moreover, time since diagnosis, treatment duration, and age were not significantly associated with sleep disorders. In general, sleep disorders are more common in the older population. Sleep status evaluated by PSQI is significantly associated with age.^[6,21,22]^ However, sleep status was not associated with age in this study, possibly because the participants were mostly relatively young. Given that aging is increasingly becoming an issue among PLWHs, the rate of sleep disorders will likely increase further in the future.

Our study also showed no association between HIV treatment regimen and CD4 levels as HIV-related factors, and sleep disorders. High or low CD4 levels have been associated with sleep disorders, although no definitive views exist.^[8,11,21,23]^ Immune statuses was also not associated with sleep disorders. Regarding therapeutic drugs, integrase inhibitors, which have been widely used in recent years, exhibit some psychological side effects.^[24]^ In our study, most of the participants (35 cases [77.8%]) used integrase inhibitors, which may explain the high rate of sleep disorders. However, the rate of sleep disorders in patients treated with integrase strand transfer inhibitors (INSTIs) was not significantly different compared with those treated with other drugs. Additionally, the psychological effects of secondgeneration INSTIs, including dolutegravir, are stronger than those of first-generation INSTIs, such as elvitegravir and raltegravir.^[25-27]^ However, we found no significant difference between the first- and second-generation INSTI users (data not shown). Thus, we could not determine whether integrase inhibitors had effects on the status of sleep in our study population.

Furthermore, complications such as hypertension, dyslipidemia, and diabetes mellitus were not associated with sleep disorders. Considering that most of our participants were relatively young, the rate of complications may have been relatively low; consequently, this study showed no association between sleep disorder and long-term complications. In general, the relationship between sleep disorder and lifestyle-related diseases has already been shown.^[28,29]^ However, in PLWHs, various factors, such as HIV itself, chronic inflammation, antiretrovirals, and stigma, may cause long-term complications, and the effect of sleep disorder on the development of long-term complications may not be significant.^[30-32]^ Further large-scale studies are needed to confirm whether sleep disorder is associated with long-term complications in PLWHs.

Our study also revealed that sleep disturbance is significantly associated with HRQOL, consistent with several studies.^[7,15]^ Maintaining a good HRQOL status has recently become one of the long-term goals of PLWHs.^[33]^ Although HIV survival has improved because of HIV treatment advancement, the HRQOL of PLWHs remains significantly lower than that of the general population, indicating a major problem.^[34]^ Sleep disorder control may be important for improving the HRQOL status of PLWHs, and sleep disorder evaluation is essential to achieve long-term goals in PLWH management.

Although this study has a single-center cross-sectional design with a small sample size, it is still clinically valuable because it reveals that sleep disorders are still common in stable PLWH with current treatment and that these disorders are an important problem in achieving future patient goals. We hope that more data will be accumulated in the future and that the actual status of sleep disorders in PLWH will be evaluated in detail to help improve HRQOL.

## Author contributions

**Conceptualization:** Yusuke Yoshino.

**Data curation:** Yusuke Yoshino, Yoshitaka Wakabayashi, Takatoshi Kitazawa.

**Investigation:** Yusuke Yoshino.

**Methodology:** Yusuke Yoshino.

**Project administration:** Yusuke Yoshino.

**Software:** Yusuke Yoshino.

**Supervision:** Takatoshi Kitazawa.

**Validation:** Yusuke Yoshino.

**Visualization:** Yusuke Yoshino.

**Writing** - **original draft:** Yusuke Yoshino.

**Writing** - **review & editing:** Yoshitaka Wakabayashi, Takatoshi Kitazawa.

## References

[1] Vitale KC Owens R Hopkins SR Malhotra A. Sleep hygiene for optimizing recovery in athletes: review and recommendations. Int J Sports Med 2019;40:535-43. 31288293 10.1055/a-0905-3103 PMC6988893

[2] Krystal AD. Psychiatric disorders and sleep. Neurol Clin 2012;30:1389-413. 23099143 10.1016/j.ncl.2012.08.018 PMC3493205

[3] Reid S Dwyer J. Insomnia in HIV infection: a systematic review of prevalence, correlates, and management. Psychosom Med 2005; 67:260-9. 15784792 10.1097/01.psy.0000151771.46127.df

[4] Mollayeva T Thurairajah P Burton K Mollayeva S Shapiro CM Colantonio A. The Pittsburgh Sleep Quality Index as a screening tool for sleep dysfunction in clinical and non-clinical samples: a systematic review and meta-analysis. Sleep Med Rev 2016;25:52-73. 26163057 10.1016/j.smrv.2015.01.009

[5] Abdu Z Dule A. Poor quality of sleep among HIV-positive persons in Ethiopia. HIV/AIDS (Auckl NZ) 2020;12:621-8. 10.2147/HIV.S279372 PMC7588272 33116924

[6] Bedaso A Abraham Y Temesgen A Mekonnen N. Quality of sleep and associated factors among people living with HIV/AIDS attending ART clinic at Hawassa University comprehensive specialized Hospital, Hawassa, SNNPR, Ethiopia. PLoS One 2020;15:e0233849. 32497153 10.1371/journal.pone.0233849 PMC7272010

[7] Faraut B Malmartel A Ghosn J, et al. Sleep disturbance and total sleep time in persons living with HIV: a cross-sectional study. AIDS Behav 2018;22:2877-87. 29855973 10.1007/s10461-018-2179-1

[8] Redman KN Karstaedt AS Scheuermaier K. Increased CD4 counts, pain and depression are correlates of lower sleep quality in treated HIV positive patients with low baseline CD4 counts. Brain Behav Immun 2018;69:548-55. 29452219 10.1016/j.bbi.2018.02.002

[9] Allavena C Guimard T Billaud E, et al. Prevalence and risk factors of sleep disturbance in a large HIV-infected adult population. AIDS Behav 2016;20:339-44. 26271816 10.1007/s10461-015-1160-5

[10] Allavena C Guimard T Billaud E, et al. Prevalence and risk factors of sleep disturbances in a large HIV-infected adult population. J Int AIDS Soc 2014;17(Suppl 3):19576. 25394083 10.7448/IAS.17.4.19576 PMC4224925

[11] Oshinaike O Akinbami A Ojelabi O Dada A Dosunmu A John Olabode S. Quality of sleep in an HIV population on antiretroviral therapy at an urban tertiary centre in Lagos, Nigeria. Neurol Res Int 2014;2014:298703. 24876959 10.1155/2014/298703 PMC4020213

[12] Dabaghzadeh F Khalili H Ghaeli P Alimadadi A. Sleep quality and its correlates in HIV positive patients who are candidates for initiation of antiretroviral therapy. Iran J Psychiatry 2013;8:160-4. 25628708 PMC4281649

[13] Wibbeler T Reichelt D Husstedt IW Evers S. Sleepiness and sleep quality in patients with HIV infection. J Psychosom Res 2012;72:439-42. 22656440 10.1016/j.jpsychores.2012.03.003

[14] Crum-Cianflone NF Roediger MP Moore DJ, et al. Prevalence and factors associated with sleep disturbances among early-treated HIV-infected persons. Clin Infect Dis 2012;54:1485-94. 22431801 10.1093/cid/cis192 PMC3334363

[15] Phillips KD Sowell RL Boyd M Dudgeon WD Hand GA. Mind-Body Research GroupSleep quality and health-related quality of life in HIV-infected African-American women of childbearing age. Qual Life Res 2005;14:959-70. 16041893 10.1007/s11136-004-2574-0

[16] Willig AL Overton ET. Metabolic consequences of HIV: pathogenic insights. Curr HIV AIDS Rep 2014;11:35-44. 24390642 10.1007/s11904-013-0191-7

[17] Kabel AM Al Thumali AM Aldowiala KA Habib RD Aljuaid SS Alharthi HA. Sleep disorders in adolescents and young adults: insights into types, relationship to obesity and high altitude and possible lines of management. Diabetes Metab Syndr 2018;12:777-81. 29673929 10.1016/j.dsx.2018.04.029

[18] St-Onge MP Grandner MA Brown D, et al. Sleep duration and quality: impact on lifestyle behaviors and cardiometabolic health: a scientific statement from the American Heart Association. Circulation 2016;134: e367-86. 27647451 10.1161/CIR.0000000000000444 PMC5567876

[19] Tokuda Y Okubo T Ohde S, et al. Assessing items on the SF-8 Japanese version for health-related quality of life: a psychometric analysis based on the nominal categories model of item response theory. Value Health 2009;12:568-73. 18783391 10.1111/j.1524-4733.2008.00449.x

[20] Chen CC Lin CY Chen YC Ko WC Li CY Ko NY. High sleep-related breathing disorders among HIV-infected patients with sleep complaints. Sleep Med 2020;75:218-24. 32861059 10.1016/j.sleep.2020.07.005

[21] Ning C Lin H Chen X, et al. Cross-sectional comparison of various sleep disturbances among sex- and age-matched HIV-infected versus HIV-uninfected individuals in China. Sleep Med 2020;65:18-25. 31706188 10.1016/j.sleep.2019.06.020

[22] Foley DJ Monjan AA Brown SL Simonsick EM Wallace RB Blazer DG. Sleep complaints among elderly persons: an epidemiologic study of three communities. Sleep 1995;18:425-32. 7481413 10.1093/sleep/18.6.425

[23] Seay JS McIntosh R Fekete EM, et al. Self-reported sleep disturbance is associated with lower CD4 count and 24-h urinary dopamine levels in ethnic minority women living with HIV. Psychoneuroendocrinology 2013;38:2647-53. 23850225 10.1016/j.psyneuen.2013.06.022 PMC3812316

[24] Hoffmann C Llibre JM. Neuropsychiatric adverse events with dolutegravir and other integrase strand transfer inhibitors. AIDS Rev 2019;21:4-10. 30899113 10.24875/AIDSRev.19000023

[25] Cid-Silva P Llibre JM Fernández-Bargiela N, et al. Clinical experience with the integrase inhibitors dolutegravir and elvitegravir in HIV-infected patients: efficacy, safety and tolerance. Basic Clin Pharmacol Toxicol 2017;121:442-6. 28627771 10.1111/bcpt.12828

[26] Lepik KJ Yip B Ulloa AC, et al. Adverse drug reactions to integrase strand transfer inhibitors. AIDS (Lond Engl) 2018;32:903-12. 10.1097/QAD.0000000000001781 29424784

[27] Peñafiel J de Lazzari E Padilla M, et al. Tolerability of integrase inhibitors in a real-life setting. J Antimicrob Chemother 2017;72:1752-9. 28333231 10.1093/jac/dkx053

[28] Knutson KL Ryden AM Mander BA Van Cauter E. Role of sleep duration and quality in the risk and severity of type 2 diabetes mellitus. Arch Intern Med 2006;166:1768-74. 16983057 10.1001/archinte.166.16.1768

[29] Kasasbeh E Chi DS Krishnaswamy G. Inflammatory aspects of sleep apnea and their cardiovascular consequences. South Med J 2006;99:58-67. 16466124 10.1097/01.smj.0000197705.99639.50

[30] Mondy K Overton ET Grubb J, et al. Metabolic syndrome in HIV-infected patients from an urban, midwestern US outpatient population. Clin Infect Dis 2007;44:726-34. 17278068 10.1086/511679 PMC3170426

[31] Palau L Menez S Rodriguez-Sanchez J, et al. HIV-associated nephropathy: links, risks and management. HIV/AIDS (Auckl NZ) 2018;10:73-81. 10.2147/HIV.S141978 PMC5975615 29872351

[32] McDonald CL Kaltman JR. Cardiovascular disease in adult and pediatric HIV/AIDS. J Am Coll Cardiol 2009;54:1185-8. 19761941 10.1016/j.jacc.2009.05.055 PMC2775806

[33] Lazarus JV Safreed-Harmon K Barton SE, et al. Beyond viral suppression of HIV - the new quality of life frontier. BMC Med 2016;14:94. 27334606 10.1186/s12916-016-0640-4 PMC4916540

[34] Miners A Phillips A Kreif N, et al. Health-related quality-of-life of people with HIV in the era of combination antiretroviral treatment: a cross-sectional comparison with the general population. Lancet HIV 2014;1:e32-40. 26423814 10.1016/S2352-3018(14)70018-9

